# Effect of Cholesterol on the Structure of a Five-Component Mitochondria-Like Phospholipid Membrane

**DOI:** 10.3390/membranes5040664

**Published:** 2015-10-30

**Authors:** Kelly Cathcart, Amit Patel, Hannah Dies, Maikel C. Rheinstädter, Cécile Fradin

**Affiliations:** 1 Department of Physics and Astronomy, McMaster University, Hamilton, ON L8S 4M1, Canada; E-Mails: kecathcart@gmail.com (K.C.); amit.69.patel@gmail.com (A.P.); 9hed@queensu.ca (H.D.); 2 Canadian Neutron Beam Centre, National Research Council, Chalk River, ON K0J 1J0, Canada; 3 Department of Biochemistry and Biomedical Sciences, McMaster University, Hamilton, ON L8N 3Z5, Canada

**Keywords:** phospholipid, cholesterol, mitochondria, cardiolipin, apoptosis, Bax, X-ray diffraction, reflectivity, wide angle X-ray scattering

## Abstract

Cellular membranes have a complex phospholipid composition that varies greatly depending on the organism, cell type and function. In spite of this complexity, most structural data available for phospholipid bilayers concern model systems containing only one or two different phospholipids. Here, we examine the effect of cholesterol on the structure of a complex membrane reflecting the lipid composition of mitochondrial membranes, with five different types of headgroups (phosphatidylcholine (PC), phosphatidylethanolamine (PE), phosphatidylinositol (PI), phosphatidylserine (PS) and cardiolipin (CL)) and a variety of hydrocarbon tails. This particular system was chosen because elevated cholesterol contents in mitochondrial membranes have been linked to a breaking down of Bax-mediated membrane permeabilization and resistance to cancer treatments. High resolution electron density profiles were determined by X-ray reflectivity, while the area per phospholipid chain, $A_{pc}$, and the chain order parameter, $S_{\text{X-ray}}$, were determined by wide-angle X-ray scattering (WAXS). We show that chain order increases upon the addition of cholesterol, resulting in both a thickening of the lipid bilayer and a reduction in the average surface area per phospholipid chain. This effect, well known as cholesterol’s condensation effect, is similar, but not as pronounced as for single-component phospholipid membranes. We conclude by discussing the relevance of these findings for the insertion of the pro-apoptotic protein Bax in mitochondrial membranes with elevated cholesterol content.

## 1. Introduction

Cellular membranes have a highly complex molecular composition, dominated by phospholipids with a large variety of headgroups, tail lengths and degrees of unsaturation [1]. All cellular membranes share common physicochemical properties, starting with the arrangement of the lipids into a bilayer with significant lateral fluidity [2,3]. Their lipid make-up, however, varies greatly depending on the type of cell or organelle they surround [4,5]. This endows each cellular membrane with unique properties (thickness, surface charge, rigidity, permeability, spontaneous curvature) adapted to their particular function. In spite of their complex lipid composition and the crucial importance of this composition for their function, our current structural understanding of cellular membranes mainly stems from experiments or simulations performed with membranes containing one, or at most two, different types of phospholipids.

The effect of cholesterol on simple membranes is a textbook example of the relationship between membrane composition and structure [6]. In one- or two-component lipid membranes, cholesterol is well known to influence both membrane thickness and chain ordering [7,8,9,10,11,12], as well as to induce the formation of cholesterol-rich liquid-ordered domains [13,14,15,16,17,18]. However, to our knowledge, no structural study has yet been performed on the effect of cholesterol on reconstituted membranes with more complex lipid compositions, to check whether it would be the same as for simple membranes.

In the cell, the question of the effect of cholesterol on membrane structure is especially crucial for mitochondrial membranes. The sterol content of healthy mitochondrial membranes is low, ∼5 mol% or less, depending on cell type [24]. Yet, higher than normal mitochondrial cholesterol levels are sometimes observed and have been linked to cancerous tumour cells and to resistance to anti-cancer therapies [25,26,27,28,29]. Several studies suggests that this could be due to the inhibition of the insertion of the pro-apoptotic protein Bax in the mitochondrial outer membrane upon an increase in the membrane cholesterol content, since this process is an essential part of the apoptotic pathway that needs to be induced during cancer treatments [30,31,32]. We, therefore, examined to what extent the effects of cholesterol observed in simple membranes can be reproduced in a multi-component phospholipid membrane, mimicking mitochondrial membranes.

**Figure 1:**
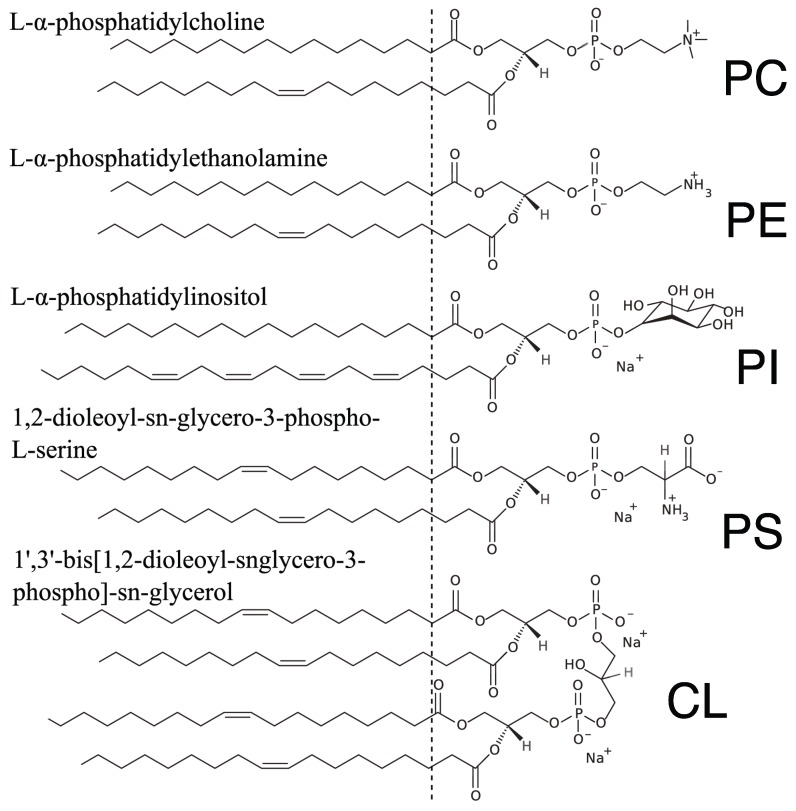
Lipid composition of the mitochondria-like membrane. A model mitochondria-like membrane was obtained by mixing naturally-extracted phosphatidylcholine (PC), naturally-extracted phosphatidylethanolamine (PE), naturally-extracted phosphatidylinositol (PI), synthetic 1,2-dioleoyl-sn-glycero-3-phospho-l-serine (DOPS) and synthetic 1’,3’-bis[1,2-dioleoyl-sn-glycero-3-phospho]-sn-glycerol (TOCL) (see Table 2 for the exact proportions). For each headgroup type, the skeletal formula of the most abundant species is represented, although for the naturally-extracted lipids, a variety of different tails are in fact present. The vertical line marks the transition from the acyl chain region to the lipid headgroup region.

The lipid composition of mitochondria resembles that of Gram-negative bacteria, in which the most abundant components are phosphatidylcholine (PC, ∼50 mol%) and phosphatidylethanolamine (PE, ∼30 mol%) [23]. Other phospholipids, such as phosphatidylinositol (PI) or phosphatidylserine (PS), are found at lower concentrations. Cardiolipin (CL), mitochondria’s signature lipid, is found at a low concentration (∼5 mol% or less) in the outer membrane and at a high concentration (up to ∼25 mol%) in the inner membrane. We therefore chose to mimic mitochondrial membranes with reconstituted lipid bilayers made of a five-component phospholipid mix, containing PC, PE, PI, PS and CL (see Table 2 for the exact composition) and a distribution of tail lengths and degrees of unsaturation. This same composition was already featured in a number of our previous studies as a model for mitochondrial membranes [19,20,21,22]. The skeletal structures of the five main phospholipid components in this membrane are shown in Figure 1.

## 2. Results

**Figure 2:**
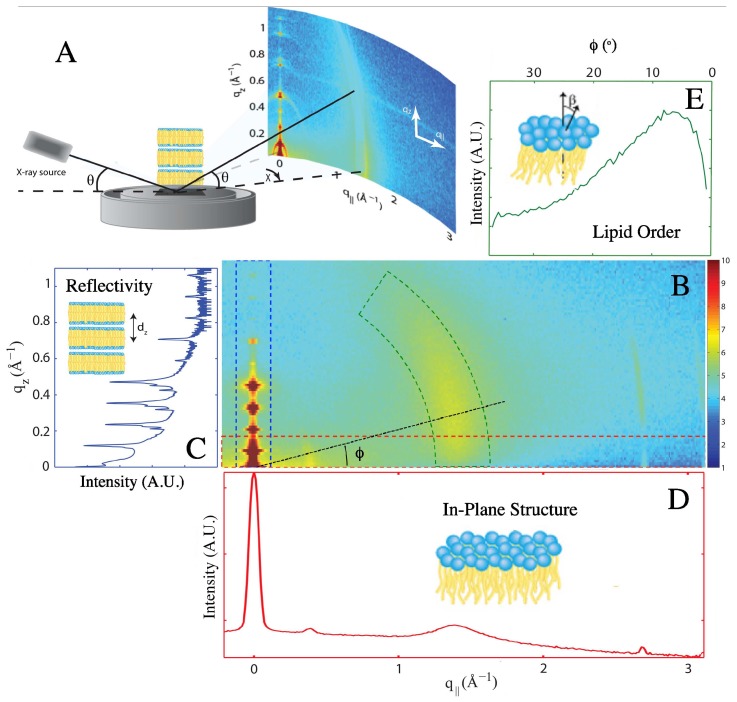
Extraction of one-dimensional scattering data from the 2D X-ray diffraction map. (**A**) Experimental X-ray diffraction set-up, showing how 2D intensity maps were recorded for stacks of highly-oriented lipid membranes. (**B**) 2D intensity map obtained for 1-palmitoyl-2-oleoyl-phosphatidylcholine (POPC) membranes. The 1D scattering data shown in (**C**,**D**,**E**) were obtained by integrating the data in the 2D map either over a small $q_{\left|\right|}$-range to obtain reflectivity curves as a function of $q_{z}$ (C), over a small $q_{z}$-range to obtain in-plane scattering curves as a function of $q_{\left|\right|}$ (D) or over a small *q*-range to obtain scattering curves as a function of *ϕ* (**E**). The regions of the 2D map used to generate each kind of 1D scattering curve are indicated by the corresponding coloured boxes.

In order to probe the molecular structure of mitochondria-like membranes, we prepared stacks of highly-oriented membranes, which we examined using two-dimensional high resolution X-ray diffraction. This allowed extracting complementary pieces of information about the bilayer structure. The bilayer thickness was inferred from the intensity of the reflectivity peaks observed in the out-of-plane scattering data. Information about the packing of the acyl chains and their ordering was obtained by looking at the position and shape of the chain-correlation peak observed in the in-plane scattering data. The precise lipid compositions of all of the membranes examined in this study are given in Table 2. The membranes were studied at *T* = 28 ${}^{\circ }$C and 83.3% ± 0.9% relative humidity (RH) to emphasize their structural features. Working at reduced RH meant that a sufficient number of higher order Bragg peaks was obtained to allow reconstructing high resolution electron density profiles [33,34]. The membrane stacks were placed inside the diffractometer, as shown in Figure 2A, and diffraction data covering a large area of reciprocal space were collected.

For a quantitative analysis of the diffracted intensity, the 2D intensity maps were cut along three different axes, as illustrated in Figure 2B. The three types of one-dimensional scattering data give access to different structural parameters, as described in detail in Section 4. The out-of-plane scattering along $q_{z}$ (reflectivity data) shows a series of Bragg peaks (Figure 2C), which can be used to reconstruct the average transverse electron density profile of the membranes [35,36,37,38,39,40]. The in-plane scattering data along $q_{\left|\right|}$ (wide-angle X-ray scattering, or WAXS; Figure 2D) contains information about the packing of the lipid acyl chains. The average surface area occupied by an acyl chain, $A_{c}$, can be determined from the position of the broad chain correlation peak observed at $q_{||,T}\approx 1.4\;{Å}^{-1}$ [39,41]. Finally, the profile of the chain-correlation peak, when plotted as a function of the polar angle *ϕ* (*i.e*., along the arc defined by the constant scattering wave vector amplitude $q=q_{T,||}$ in the $\left(q_{\left|\right|},q_{z}\right)$ plane; Figure 2E) allows estimating an orientational order parameter for the lipids’ carbon chains. The more disordered the lipid acyl chains, the broader the chain correlation peak [42]. Note that two additional peaks are seen in the in-plane scattering data that are not related to the stacked membrane structure: the Kapton window of the hydration chamber leads to scattering at $q_{\left|\right|}\approx$ 0.4 Å${}^{-1}$, while the silicon substrate leads to scattering at $q_{\left|\right|}\approx 2.75\;{Å}^{-1}$ and $q_{z}\approx 0.5\;{Å}^{-1}$.

### 2.1. Structure of Multi-Component Mitochondria-Like Membranes

We compared the structure of the five-component mitochondria-like membrane to that of a simple one-component POPC (1-palmitoyl-2-oleoyl-phosphatidylcholine) membrane. POPC was chosen as a control, since PC is the most abundant phospholipid headgroup in the mitochondrial-like membrane and since 16:0/18:1 (1-palmitoyl-2-oleoyl) is the most abundant chain composition in the naturally-extracted PC used to form that membrane. In addition, POPC has often been used as a reference system to study lipid membrane properties [43,44,45,46,47,48,49].

For the membranes studied here, the wide-angle scattering data always showed a single broad chain correlation peak at $q_{T,||}∼$1.4 Å${}^{-1}$, with a distinctive vertical rod-like shape typical of diffraction obtained for 2D systems (Figure 2B,D). A broad peak is indicative of a $L_{\alpha }$ fluid liquid crystalline phase (also referred to as liquid disordered phase, $L_{d}$) [42]. From the position of the chain correlation peaks, we found that the area per chain was comparable in both membranes: $A_{c}=23.4\;{Å}^{2}$ in the POPC membrane and $A_{c}=23.3\;{Å}^{2}$ in the mitochondria-like membrane (see Table 1 for a list of the structural parameters measured for both membranes).

**Table 1:** Structural properties measured for the samples used in this study.

Sample	$A_{c}({Å}^{2})$	$A_{\mathrm{pc}}({Å}^{2})$	$S_{\text{X-ray}}$	$d_{z}$ (Å)	$d_{\mathrm{hh}}$ (Å)	$w_{h}$(Å)	$w_{c}$(Å)
POPC	23.40 ± 0.04	23.4	0.36	53.2 ± 0.8	39.1 ± 0.5	9.7	7.7
Mitochondria-like	23.31 ± 0.06	23.3	0.26	50.3 ± 0.2	39.9 ± 0.5	8.5	6.7
Mitochondria-like (10% cholesterol )	24.1 ± 0.2	23.2	0.39	50.7 ± 0.2	40.3 ± 0.5	9.0	7.6
Mitochondria-like (20% cholesterol)	24.6 ± 0.1	22.8	0.54	51.4 ± 0.3	42.6 ± 0.5	9.9	10.2
Mitochondria-like (30% cholesterol)	24.8 ± 0.2	21.8	0.48	53.9 ± 0.7	43.1± 0.5	10.8	10.6

In a fluid phase, lipids are not expected to have a favoured tilt angle with respect to the membrane normal, and therefore, the chain correlation peak profile should be centred at ϕ=0. However, as the intensity scattered parallel to the plane of the membrane ($\phi ≲10^{\circ }$ values) is obstructed by the membrane itself, the peak maximum was not observed (see Figure 2E). Therefore, only the intensity obtained for $\phi ⩾13^{\circ }$ was considered when examining the profile of the chain correlation peak. Assuming a Maier–Saupe orientational distribution function for the chains leads to an analytical expression for the peak scattering intensity as a function of the polar angle, I(ϕ), which is dependant on the width of the chain orientation distribution (Equation (4)) [42]. The width of this distribution can in turn be related to the chain orientational order parameter, $S_{\text{X-ray}}$, as described in more detail in Section 4.4. For the monounsaturated POPC membrane, we found that $S_{\text{X-ray}}=0.36$. Strikingly, the order parameter measured for the mitochondria-like membrane, which contains a significant fraction of di-monounsaturated and polyunsaturated chains (see Figure 1), is significantly smaller: $S_{\text{X-ray}}=0.26$.

**Figure 3:**
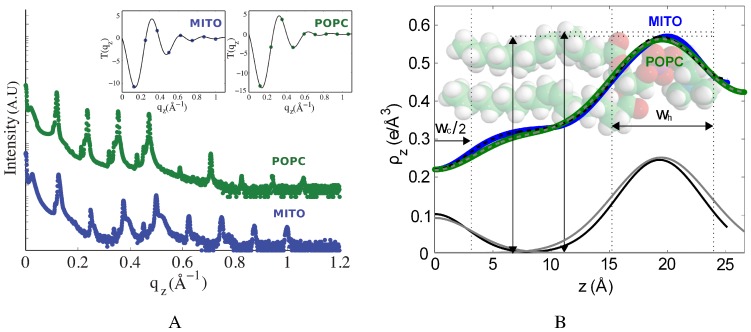
Electron density profiles of the mitochondria-like and POPC membranes. (**A**) The reflectivity curves for the mitochondria-like and POPC membranes (the latter was shifted upwards for clarity) show eight and nine well-developed Bragg peaks, respectively. The insets show the fits of the peaks amplitude with the continuous $T(q_{z})$ function, plotted for the best combination of phases, $\nu_{n}$. (**B**) The electron density profiles of the mitochondria-like and POPC membranes were determined from the data shown in (A) and modelled with Gaussian peak profiles to represent different lipid molecular components, as explained in the text. The Gaussian components of the fit corresponding to the headgroup region and the chain terminal $CH_{3}$ region are represented in black and grey for the mitochondria-like and POPC membranes, respectively. Dashed lines represent the result of the fit. The FWHMs of the headgroup region, $w_{h}$, and chain terminal region, $w_{c}$, are indicated on the figure, and their values are listed in Table 1.

The reflectivity curves obtained for the POPC and the mitochondria-like membranes are compared in Figure 3A. Nine pronounced Bragg peaks were observed for the former and eight for the latter, indicative in both cases of a well-ordered lamellar structure. The lamellar spacing, determined from the position of the Bragg peaks, was found to be $d_{z}$ = 53.2 Å for the POPC membrane stack. The lamellar spacing of the mitochondria-like membrane was slightly lower, $d_{z}$ = 50.3 Å.

The average electron density profile, $\rho_{z}$, was calculated by Fourier transformation of the integrated peak intensities, as outlined in Section 4.5. The electron density profiles obtained for the POPC and the mitochondria-like membranes are shown in Figure 3B. Both are consistent with the electron density profiles of lipid bilayers in the liquid-disordered state. The maxima in electron density on either side of the membranes correspond to the electron-rich phosphorous group found in the headgroup region. The membrane thickness, $d_{hh}$, defined here as the distance between these two maxima, was found to be $d_{hh}=39.1\;Å$ for the POPC membrane (slightly larger than that of a fully-hydrated POPC membrane, $d_{hh}=37\;Å$ [43]) and $d_{hh}=39.9\;Å$ for the mitochondria-like membrane.

In order to further characterize the electron density profiles, they were fit using Gaussian functions to represent the average electron density contributed by different components. This has been shown in simulations to provide a good description of a fluid membrane [50] and has often been used to interpret membrane diffraction data [35,36,43]. We used a single Gaussian peak to represent the headgroup region and its associated water molecules and an inverted Gaussian peak to represent the chains terminal $CH_{3}$ region in the centre of the membrane, as done previously in [43]. These Gaussian functions were set on top of a constant baseline whose value then represents the acyl chain electron density in a dehydrated membrane, $\rho_{CH_{2}}$. Since the studied membranes were not fully hydrated and therefore did not necessarily have a significant layer of pure bulk water between bilayers in the stack, no specific peak was attributed to a water layer, and instead, for each headgroup, we included a Gaussian with the same amplitude and width, centred at $2d-z_{h}$ to represent the headgroup region of the neighbouring bilayer. The equation used to fit the electron density profile thus was:

(1) $$\rho_{z}\left(z\right)=\rho_{CH_{2}}-H_{c}e^{-\frac{z^{2}}{2w_{c}^{2}}}+H_{h}\left(e^{-\frac{{\left(z-z_{h}\right)}^{2}}{2w_{h}^{2}}}+e^{-\frac{{\left(z-\left(2d_{z}-z_{h}\right)\right)}^{2}}{2w_{h}^{2}}}\right)$$

In the above, $H_{h}$ and $z_{h}$ are the amplitude and position of the Gaussian representing the headgroup region. $H_{c}$ is the amplitude of the Gaussian representing the terminal acyl chain region. $w_{h}$ and $w_{c}$ are the full width at half maximum (FWHM) of the Gaussians representing the headgroup and terminal acyl chain regions, respectively.

The electron density profiles of both the POPC and the mitochondria-like membranes were fit with Equation (1), as shown in Figure 3B, and the results of these fits are presented in Table 1. In a previous study, where several types of diffraction measurements were used to achieve high resolution, the headgroup region of simple one-component lipid bilayers has been found to be best approximated by two Gaussians, one for the phosphatidylcholine region and one for the carbonyl and glycerol region [43]. However, in the case of the POPC membrane studied here, the use of two headgroup Gaussian peaks was found to lead to over fitting. In the case of the mitochondria-like membrane, using two Gaussian for the headgroup region did give a better fit, probably because of the presence of lipids with various headgroups. For consistency, the fits shown in Figure 3B and the results shown in Table 1 were those obtained using a single Gaussian peak.

### 2.2. The Effect of Cholesterol on Mitochondria-Like Membranes

To study the effect of cholesterol on complex membranes, mitochondria-like membranes with various amounts of cholesterol were prepared, with compositions listed in Table 2. In order to maintain the overall surface charge and spontaneous curvature of the membrane, the concentration of neutral PC lipids was decreased as cholesterol concentration was increased.

**Figure 4:**
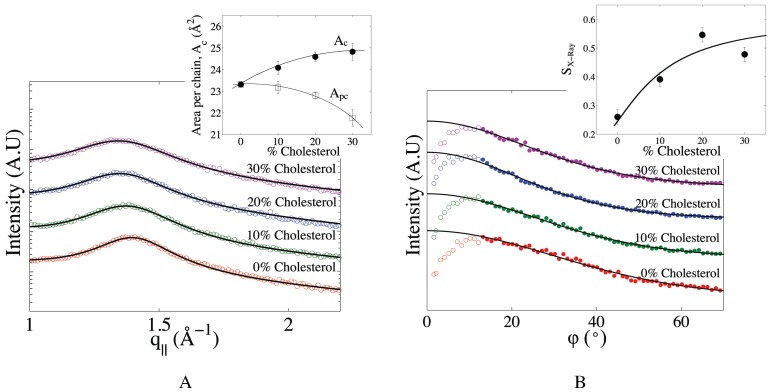
Effect of cholesterol on the packing and orientational order of mitochondria-like membranes. (**A**) Chain correlation peaks for mitochondria-like membranes containing between 0 and 30 mol% cholesterol. Each curve is fit with a Gaussian profile on a slopping background (solid lines). Curves are shifted vertically for clarity. Inset: average area per chain (*i.e*., including cholesterol chains), $A_{c}$, directly inferred from the position of the peak (filled symbols), compared to the average area per phospholipid chain, $A_{pc}$, as corrected for cholesterol volume using Equation (2) (open symbols). Solid lines are a guide for the eyes. (**B**) Integrated intensities of the chain correlation peak as a function of polar angle. Curves are shifted vertically for clarity. Continuous lines represent fits of the filled symbol data with Equation (4), while the open symbols were not included in the fit. Inset: X-ray chain orientational order parameter, $S_{\text{X-ray}}$, calculated from that fit.

The average area per chain (which includes the “chain” of cholesterol molecules), $A_{c}$, was determined from the position of the chain correlation peak in the in-plane diffraction data. The shift of that peak to smaller $q_{\left|\right|}$-values with increasing cholesterol content reflects an increase of the average chain-chain distance and an increase of the corresponding average area per chain (Figure 4A). At the same time, the broadening of the peak with cholesterol addition points to a larger average distribution of chain-chain distances. This can be attributed to the fact that in the presence of cholesterol, phospholipid chains can now have two sorts of neighbours, other phospholipid chains or cholesterol chains. The latter interaction is characterized by a slightly larger distance. To specifically examine the influence of cholesterol on the organization of the phospholipid chains, we expressed the average area per chain as a function of the average area per phospholipid chain, $A_{pc}$, and the area per cholesterol chain, $A_{chol}$, as proposed in [51]:

(2) $$A_{c}\left(x\right)=\left(1-x\right)A_{pc}\left(x\right)+xA_{chol}$$

where *x* is the cholesterol fraction and where it is assumed that $A_{chol}$= 39 Å${}^{2}$ is a constant [52]. Equation (2) can then be used to calculate $A_{pc}\left(x\right)$. As can be seen in the inset in Figure 4A, whereas $A_{c}$ increases with cholesterol content, $A_{pc}$ markedly decreases. This confirms that cholesterol’s condensing effect is present in multi-component mitochondria-like membranes.

The influence of cholesterol on lipid tail order was further assessed by extracting the order parameter, $S_{\text{X-ray}}$, from the wide-angle peak profiles shown in Figure 4B. As expected, $S_{\text{X-ray}}$ increases with cholesterol content (see the inset in Figure 4B). This behaviour is comparable to that observed for single-component membranes, with a sharp increase of $S_{\text{X-ray}}$ for cholesterol contents between 0% and 20%, then a plateauing of the chain order around 30 mol% cholesterol, at a value of $S_{\text{X-ray}}$, about double that obtained at 0% cholesterol [42,53].

**Figure 5:**
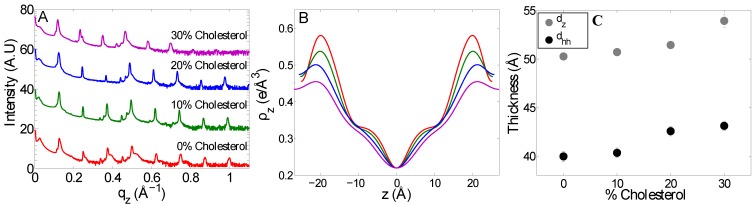
Effect of cholesterol on the lamellar spacing and thickness of mitochondria-like membranes. (**A**) Reflectivity curves for mitochondria-like membranes with 0, 10, 20 and 30 mol% cholesterol. The curves are shifted vertically for clarity. Intensity is plotted on a logarithmic scale. (**B**) Scaled electron density profiles of mitochondria-like membranes with the same cholesterol content as in (A). (**C**) Lamellar spacing, $d_{z}$, and peak-to-peak membrane thickness, $d_{hh}$, as a function of cholesterol content.

The reflectivity data for these membranes is shown in Figure 5A. All systems were found to form lamellar structures with pronounced Bragg reflections. The addition of cholesterol seems to have an effect on the topology of the membrane stacks, as the number of diffraction orders passes from eight to six when the amount of cholesterol reaches 30 mol%. The corresponding electron density profiles are shown in Figure 5B. These profiles were fit with the Gaussian model described in Section 2.1. With the exception of the profile obtained in the absence of cholesterol, they were well fit by a single Gaussian peak to describe the headgroup region. The results of these fits are shown in Table 1. The electron density in the headgroup region decreased with increasing cholesterol content as more electron-rich lipid molecules are replaced by smaller cholesterol molecules. At the same time, the headgroup peak shifts further from the centre of the membrane, indicative of an increase in the bilayer thickness, $d_{hh}$, which coincides with the increase in lamellar spacing, $d_{z}$, as shown in Figure 5C.

## 3. Discussion

### 3.1. How Different Is the Multi-Component Mitochondria-Like Membrane from a POPC Membrane?

In the conditions chosen for our experiments (83.3% RH, T=28${}^{\circ }$C), both the mitochondria-like membrane and the POPC membrane were in the liquid-disordered, fluid $L_{\alpha }$ phase. A fluid phase was expected for the POPC membrane, even at this relatively low RH, since the gel phase to fluid phase transition for POPC is T=-3.7${}^{\circ }$C [44,54]. It was also expected for the mitochondria-like membrane, since at least three of its lipid components have a transition temperature below room temperature [55] and since diffusion experiments have shown liquid-like diffusion of lipids in the same system at full hydration [22,56]. Yet, although they were in the same phase, the structure of these two membranes differed in a number of ways.

The mitochondria-like membrane was slightly (≃1Å) thicker than the POPC membrane. This difference can simply be attributed to the fact that some of its lipid components had hydrocarbon chains longer than that of POPC (see Figure 1), since membrane thickness strongly correlates with the length of hydrocarbon chains [12]. At the same time, the lamellar spacing was significantly (≃3Å) thinner for the mitochondria-like membrane. Thus, the water layer found between mitochondria-like membranes was thinner than that found between POPC membranes. The fact that only a thin water layer is present in the case of the mitochondria-like membrane could be due to favourable interactions between the negatively-charged lipids in the membrane and cations in solution. Electrostatic interactions would be reduced for the overly neutral POPC membrane, and therefore, we would expect to see a slightly thicker water layer in that case.

The most notable difference between the mitochondria-like and POPC membranes is the higher degree of chain disorder in the multi-component membrane, as reflected in the values of the chain order parameter, $S_{\text{X-ray}}$, measured by WAXS. It has been shown that $S_{\text{X-ray}}$ only differs from the chain order parameter measured by NMR by a multiplication factor [42]. It has also been pointed out that for membranes containing unsaturated chains, as was the case for the mitochondria-like membrane we studied, $S_{\text{X-ray}}$ gives a more consistent evaluation of the chain order [53]. As predicted by coarse-grained simulations [57], the value of $S_{\text{X-ray}}$ for the monounsaturated POPC membrane falls between those measured by the same method for fully-hydrated membranes of the saturated lipid DPPC (1,2-dipalmitoyl PC, or PC(16:0/16:0)) and of the di-monounsaturated lipid DOPC (1,2-dioleoyl PC, or PC(18:1/18:1)) [42]. On the other hand, it is close to that measured for fully-hydrated membranes of the mono-unsaturated SOPC (1-stearoyl-2-oleoyl PC, or PC(18:0/18:1)) [53]. This confirms that, for one-component membranes, the degree of unsaturation of the membrane is a determinant factor of chain disorder. The mitochondria-like membrane was, like a real membrane, formed of phospholipids with a large distribution of chain lengths and degrees of unsaturation (40% saturated chains, 40% mono-unsaturated chains, 20% polyunsaturated chains, *i.e.*, 0.8 unsaturated bonds per chain on average). According to our findings (see Table 1), and unsurprisingly, the chain order parameter of the mitochondria-like membrane is 30% lower than that of the POPC membrane (50% saturated chains, 50% mono-unsaturated chains, *i.e.*, 0.5 unsaturated bonds per chain on average). On the other hand, and more surprisingly, it is about the same as that measured for membranes formed with DOPC (100% mono-unsaturated chains, *i.e.*, one unsaturated bond per chain) [53]. This suggests that a membrane consisting of lipids with a number of different chains may have a lower order parameter than membranes composed uniquely of any of its components.

Finally, we note that although $S_{\text{X-ray}}$ is notably lower for the mitochondria-like membrane than for the POPC membrane, the area per chain is almost the same for both membranes, and lower than for a fully-hydrated POPC membrane, $A_{c}=34\;{Å}^{2}$ [43], but instead close to that obtained for optimum packing of acyl chains, $A_{c}≃20\;{Å}^{2}$ [58]. Thus, although the chains in the mitochondria-like membrane are relatively disordered, the packing in the headgroup region is close to ideal.

### 3.2. Effect of Cholesterol on the Multi-Component Mitochondria-Like Membrane

High cholesterol concentrations in the plasma membrane of mammalian cells are known to decrease membrane fluidity and increase membrane rigidity and thickness, thereby providing a stronger cellular enclosure. In simple, one-component membranes, the effect of cholesterol has been quantitatively studied by many different methods (experimental and simulations, see e.g., [6] for a review), in particular for POPC membranes [45]. Cholesterol has been shown to thicken the membrane and simultaneously (since the volume of the hydrophobic region of the membrane is conserved [59]) to reduce the surface area allotted to each phospholipid, a process known as the condensing effect [60]. This occurs as a result of a decrease in chain disorder, thought to be due to cholesterol’s interaction with the acyl tails, resulting in a straightening out of the lipid chains and a reduction in their fluidity [61,62]. Finally, cholesterol is known to segregate and form highly-ordered domains (the equivalent of lipid rafts in cells) [6,16].

The orientational order parameter of the hydrocarbon chains in the mitochondria-like membranes quickly doubles upon an increase in cholesterol content (Figure 4B), approaching a plateau similar to what has been observed for single-component membranes [53]. However, $S_{\text{X-ray}}$ only reaches a value of ≈0.5 at 30 mol% cholesterol, which is lower than what was observed at a high cholesterol content for di-monounsaturated lipids (e.g., DOPC) and higher hydration levels [53]. This is strong evidence that cholesterol’s ordering effect is reduced in multi-component membranes.

Concurrently, with the increase in chain ordering, the thickness of the mitochondria-like membrane increased by 8%, and the surface area per phospholipid chain accordingly decreased by 6% after the addition of 30% cholesterol. This condensing effect is weaker than that observed for single-component membranes, where the area condensation is shown to be around 10%–20% for 30% cholesterol (in experiments and simulations done at a slightly higher RH) [62,63]. Our data, again, suggest that the ordering brought about by cholesterol is not as complete in the five-component membrane as in homogeneous one-component membranes.

Cholesterol also affects the lipid headgroup region. As the membrane thickness increases, so does the lamellar spacing (see Figure 5). This implies that the thickness and therefore structure of the water layer around the phospholipid headgroups do not significantly change upon the addition of cholesterol. On the other hand, we observed that the electron density in the headgroup region strongly decreases upon cholesterol addition (Figure 5; by 20%). One contributing factor to this decrease in average headgroup electron density must be that cholesterol’s polar region is small (just one hydroxyl group). In addition, the width of the headgroup region, $w_{h}$, increases (see Table 1; by 20%). The overall decrease in headgroup electron density has implications for the distribution of ions in the vicinity of the membrane surface and the type of electric potential profile encountered by molecules approaching the membrane, such as proteins.

Our data suggest that the five-component mitochondria-like membranes studied here were all below the cholesterol solubility limit (usually found around 40 mol% cholesterol [63,64,65]). In addition, we did not find any evidence of the formation of lipid domains differentiated by lipid chain order upon the addition of cholesterol. This is consistent with atomic force microscopy imaging of single supported lipid bilayers with the exact same lipid composition, which showed no lateral heterogeneity at the ≃100 nm scale or above [22].

Overall, we found that cholesterol affected the mitochondria-like membranes in a similar fashion to one and two-component membranes; by increasing the membrane thickness and decreasing the area per phospholipid chain. However, the ordering brought about by cholesterol was found to be less complete than in homogeneous single-component membranes.

### 3.3. Implications for Mitochondria with Higher than Normal Cholesterol Content

In healthy cells, mitochondrial membranes contain only a minimal amount of cholesterol. This, however, can change in different disease states, including cancer [26,27,28]. In particular, increased concentrations of cholesterol in the mitochondria membrane have been shown to correlate with chemotherapy resistance [29], leading to the idea that targeting cholesterol production should be explored as a cancer treatment option [66]. A leading hypothesis is that mitochondrial cholesterol affects cancer treatment outcome by blocking outer membrane permeabilization during apoptosis. This is supported by several biophysical studies that have shown that, *in vitro*, cholesterol can block the pore formation activity of the pro-apoptotic protein Bax in mitochondria-like membranes [30,31,32]. Interestingly, all of these studies concur that the step that is specifically blocked by the presence of cholesterol in the membrane is the insertion of the protein in the lipid bilayer (as opposed to membrane docking, binding to other membrane proteins or pore formation itself). This effect is non-specific, as it can be replicated with the enantiomer of cholesterol [31]. It is thus likely brought about by changes in the membrane physical properties.

Our study suggests three possible explanations for the inhibition of Bax membrane insertion upon the addition of cholesterol. The first possible cause for this inhibition is the observed changes in the headgroup region, namely the reduction in area per phospholipid headgroup and the decrease in electron density in the headgroup region. The former would result in the elimination of defects in the headgroup region, which may be required for Bax insertion into the membrane, and the latter could change how Bax “sees” the membrane as it approaches and orients itself with respect to it. A second possible cause for the inhibition of Bax insertion is the observed increase in chain order ($S_{\text{X-ray}}$). Chain ordering has been shown to affect both the bending and compressibility of a membrane [67]. A decrease in bending flexibility might prevent lipidic pore formation, while a decrease in lateral compressibility might decrease lateral density fluctuations, which may be indispensable for Bax insertion. A third possibility is that by increasing membrane thickness, cholesterol makes it difficult for Bax to adopt a transmembrane configuration. The hydrophobic part of the two pore-forming *α*-helices of Bax are about 15 residues long [68], *i.e.*, about 23 Å long. The hydrophobic part of the mitochondria-like model membrane is about 30 Å (this value is obtained by subtracting the headgroup width, ∼10 Å, from the peak-to-peak thickness listed in Table 1). This suggests that there might be a small hydrophobic mismatch between the two pore-forming helices of Bax and the slightly larger hydrophobic part of the mitochondrial membrane (this is also suggested by the fact that Bax insertion in mitochondria-like membranes causes membrane thinning [20]). Further thickening of the membrane by 3 Å due to cholesterol addition would make this mismatch even larger and, thus, Bax insertion less favourable. Follow up studies will be required in order to see which of these effects is most relevant to the interaction with the protein.

## 4. Experimental Section

### 4.1. Lipids and Reagents

Naturally-extracted l-*α*-phosphatidylcholine (egg PC, Catalogue Number 840051), naturally-extracted l-*α*-phosphatidylethanolamine (egg PE, Catalogue Number 841118), naturally-extracted l-*α*-phosphatidyl-inositol (liver PI, Catalogue Number 840042), 1,2-dioleoyl-sn-glycero-3-phospho-l-serine (DOPS, Catalogue Number 840035) and 1’,3’-bis[1,2-dioleoyl-sn-glycero-3-phospho]-sn-glycerol (TOCL, Catalogue Number 710335) were purchased from Avanti Polar Lipids (Alabaster, AL, USA). Cholesterol was purchased from Sigma Aldrich (Mississauga, ON, Canada).

**Table 2:** Composition of the membranes examined in this study. For each lipid composition, the estimated number of water molecules per phospholipid headgroup, $n_{W}$, as well as the average number of electron per chain, $\langle e_{c}\rangle$, calculated with Equation (10), as explained in Section 4.5, are indicated.

Sample	POPC (mol%)	Egg PC (mol%)	Egg PE (mol%)	Liver PI (mol%)	DOPS (mol%)	TOCL (mol%)	Cholesterol (mol%)	$n_{W}$	$\langle e_{c}\rangle$
POPC	100	–	–	–	–	–	–	7	245
Mitochondria-like	–	48	28	10	10	4	0	4.5	235.4
Mitochondria-like	–	38	28	10	10	4	10	4.5	235.9
10% cholesterol									
Mitochondria-like	–	28	28	10	10	4	20	4.5	236.4
20% cholesterol									
Mitochondria-like	–	18	28	10	10	4	30	4.5	236.9
30% cholesterol									

### 4.2. Preparation of Highly-Oriented Membrane Stacks

Highly-oriented multi-lamellar lipid membranes with amounts of cholesterol varying between 0 and 30 mol% (see Table 2 for exact lipid compositions) were prepared on silicon wafers using the rock-and-roll method [69], as previously described in detail [39,40,65]. Single-sided polished 300 μm-thick silicon (100) wafers (purchased from Silchem, Germany) were pre-cut into 1 × 1 cm${}^{2}$ chips and cleaned by sonication in dichloromethane (DCM) at 37 ${}^{\circ }$C for 25 min to remove all organic contamination. This step also leaves the substrates in a hydrophobic state. Each wafer was thoroughly rinsed three times by alternating between ∼50 mL of ultra pure water and methanol. The wafers were then dried under a stream of nitrogen gas and placed on a heating block set to 40 ${}^{\circ }$C. Lipids and cholesterol were mixed in the right proportion in chloroform, after which the solvent was left to evaporate under a stream of argon gas for one hour and then in a vacuum at 25 ${}^{\circ }$C for 12 h. The lipid mixture was then re-dissolved in a 1:1 chloroform:2,2,2-trifluoroethanol (TFE, purchased from Sigma Aldrich) solution at a total concentration of 15 mg/mL. Both the prepared lipid solution and cleaned silicon wafers were allowed to equilibrate for 30 min at 40 ${}^{\circ }$C in a tilting incubator (VWR Rotating Incubating), after which 70 μm of lipid solution was deposited on each substrate and left in the incubator for 10 min in order for the solvent to evaporate. The tilt and speed of the incubator were set to 1 and 20, respectively, to allow the lipids to evenly coat the surface of the substrate. The samples were then placed inside a vacuum at 37 ${}^{\circ }$C for 12 h to remove all traces of solvent. One day prior to the X-ray experiment, the samples were re-hydrated and annealed to remove potential defects in the bilayers. This process was performed in a sealed hydration chamber placed in an incubator at 30 ${}^{\circ }$C for 12 h. The hydration chamber contained pure water to provide 100% RH. This procedure results in highly-oriented multi-lamellar membrane stacks and a uniform coverage of the silicon substrates. Samples were then stored at room temperature in an inert environment of nitrogen gas to avoid oxidation of the unsaturated lipids.

### 4.3. X-Ray Scattering Experiments

X-ray scattering data were collected using the biological large angle diffraction experiment (BLADE) in the Laboratory for Membrane and Protein Dynamics at McMaster University. BLADE operates a 9 kW (45 kV, 200 mA) CuK*α* rotating anode, which produces X-rays with a wavelength of *λ* = 1.5418 Å, which are focused to form a parallel, monochromatic beam with intensity up to $10^{10}$ counts/mm${}^{2}$/s. The source and detector arms of BLADE are both mobile, allowing for the sample to remain horizontal (as depicted in Figure 2).

Samples were placed inside of a humidity chamber equipped with Kapton windows to control the temperature and hydration of the membrane samples. Humidity was controlled by placing saturated KCl solutions inside the chamber. Samples were left to equilibrate for 13 h at 28 ${}^{\circ }$C in 83.3% ± 0.9% RH. Once equilibrium was reached, a two-dimensional intensity map of reciprocal space for 0.03 Å${}^{-1}<q_{z}<$1.1 Å${}^{-1}$ and 0 Å${}^{-1}<q_{\left|\right|}<$3.1 Å${}^{-1}$ was collected. One-dimensional scattering data were extracted from this map and used to determine the membrane structure as explained below.

### 4.4. Chain Packing and Chain Order Parameter

The acyl chain correlation peak, found around $q_{\left|\right|}\approx 1.4\;$Å${}^{-1}$, was used to determine both the average area per chain and the chain order parameter. The in-plane peak profile as a function of $q_{\left|\right|}$ was first generated by integrating the scattering data over a $q_{z}$ range of 0 Å${}^{-1}<q_{z}<$ 0.3 Å${}^{-1}$. The peak position, $q_{T,||}$, was determined by fitting this profile with a Gaussian peak profile and a linear background. The average area per chain, $A_{c}$, was then calculated assuming the lipid tails form a densely-packed structure with local hexagonal symmetry (planar group p6, as shown, for instance, in [16]), which leads to [39,42]:

(3) $$A_{c}=\frac{8\pi^{2}}{\sqrt{3}q_{T,||}^{2}}$$

The chain order parameter, $S_{\text{X-ray}}$, was determined using the out-of-plane peak profile as a function of the polar angle *ϕ*, as defined in Figure 2, following the method outlined in [42,70]. The out-of-plane peak profile was obtained by integrating the scattering data over a small *q* range (1.15 Å${}^{-1}<q<1.55\;$Å${}^{-1}$) as illustrated in Figure 2B. Assuming that the lipid chains can be assimilated to straight rods with a distribution of tilt angles that follows a Maier–Saupe distribution, $f\left(\beta \right)\propto e^{m\cos^{2}\beta }$, the out-of-plane chain correlation peak was fit with the following expression [42]:

(4) $$I\left(\phi \right)=I_{\text{b}}+\frac{C}{8}\frac{\sqrt{m}}{e^{m}D\left(\sqrt{m}\right)}e^{\frac{m\cos^{2}\phi }{2}}I_{0}\left(\frac{m\cos^{2}\phi }{2}\right)$$

In the above, $I_{0}$ is the modified Bessel function of the first kind, *D* is Dawson’s integral, $I_{\text{b}}$ is the background intensity and *C* is a constant accounting for the amount of sample contributing to the scattering, beam intensity and exposure time. The parameter *m* describing the width of the chain angular distribution is directly related to the chain orientational order parameter, $S_{\text{X-ray}}=\frac{1}{2}\left(3<cos^{2}\beta >-1\right)$, through [42]:

(5) $$S_{\text{X-ray}}=\frac{1}{2}\left(3\frac{\int_{0}^{\pi }2\pi \sin\beta \cos^{2}\beta e^{m\cos^{2}\beta }d\beta }{\int_{0}^{\pi }2\pi \sin\beta e^{m\cos^{2}\beta }d\beta }-1\right)$$

### 4.5. Electron Density Profile

The electron density of the membrane was calculated though Fourier transformation of the reflectivity data according to the protocol already outlined in [34,39,40]. Briefly, $I_{n}$ was obtained by integrating the intensity under the peak and subtracting a linear background. The lamellar spacing, $d_{z}$, was determined from the position of the Bragg peaks as the average of $2\pi n/q_{n}$. The unscaled electron density profile of the membrane, $\rho_{u}\left(z\right)$, was then calculated using the one-dimensional Fourier transform:

(6) $$\begin{array}{ccc} \rho_{u}\left(z\right) & = & \frac{2}{d_{z}}\sum_{n=1}^{N}\nu_{n}\sqrt{I_{n}q_{n}}\cos\left(\frac{2\pi n}{d_{z}}z\right) \end{array}$$

*N* is the order of the highest order Bragg peak observed in the reflectivity data. The peak intensities, $I_{n}$, are multiplied by $q_{n}$ (Lorentz correction [71]) to obtain the value of the form factor at that position, $F\left(q_{n}\right)=\nu_{n}\sqrt{I_{n}q_{n}}$. Due to the centro-symmetry of the samples, the complex bilayer form factor is reduced to a real quantity with phases $\nu_{n}=\pm 1$. The phases were assessed by fitting the experimentally-obtained values of the form factor with the continuous function [72]:

(7) $$T\left(q_{z}\right)=\sum_{n}\nu_{n}\sqrt{I_{n}q_{n}}\text{sinc}\left(\left[d_{z}q_{z}-n\right]\right)$$

An example is shown in the insets of Figure 3A. For all samples, the best fit was achieved for the set of phases $\nu_{n}=\left[-1,-1,+1,-1,+1,-1,+1,-1\right]$, except for the 30% cholesterol sample for which $\nu_{n}=\left[-1,-1,+1,-1,+1,+1,-1,+1\right]$.

In order to put the electron density profiles determined by Equation (6) on an absolute electron density scale, the following scaling was used:

(8) $$\rho \left(z\right)=a+b\rho_{u}\left(z\right)$$

The two scaling parameters were found assuming that the electron density in the centre of the lipid bilayer was equal to the density of a methyl group, $\rho_{{\text{CH}}_{3}}=0.22\;e/\;{Å}^{3}$, and that the integrated electron density over a single leaflet of the bilayer must be equal to the average number of electrons per surface area expected given the known bilayer lipid composition and the measured area per chain, $A_{c}$:

(9) $$\int_{0}^{d_{z}/2}\rho_{s}dz=\frac{\langle e_{c}\rangle }{A_{c}}$$

The number of electrons per chain for each lipid, $e_{c,i}$, is approximated to be the total number of electrons per lipid, $e_{l,i}$, divided by the number of tails belonging to that lipid $n_{c,i}$, such that $e_{c,i}=e_{l,i}/n_{c,i}$; where for PC, PE, PI and DOPS, $n_{c,i}=2$, for TOCL $n_{c,TOCL}=4$ and for cholesterol $n_{c,CHOL}=1$. The average number of electrons per chain for a given sample, $\langle e_{c}\rangle$, listed in Table 2, was then determined by a weighted sum of $e_{c,i}$ for a given sample membrane.

(10) $$\langle e_{c}\rangle =\sum_{i}\left(\frac{M_{i}n_{c,i}}{\sum_{i}M_{i}n_{c,i}}e_{c_{i}}\right)+\frac{n_{W}e_{W}}{2}$$

where $M_{i}$ and $n_{c,i}$ are the molar fraction and the number of tails for lipid *i*, respectively. Values of $M_{i}$ for each sample mixture are listed in Table 2. The average number of water molecules associated with each phospholipid headgroup in a given sample membrane is $n_{W}$, and the number of electrons per water molecule is $e_{W}=10$. Thus, $n_{W}e_{W}/2$ is the average number of electrons contributed by water molecules per chain. Since about seven water molecules associate on average with each POPC headgroup at 83% RH [47], we considered here that $n_{W}e_{W}/2=35$ electrons contributed by water molecules were associated on average with each phospholipid chain in the POPC membrane. For the mitochondria-like membranes, however, it was found that the water layer associated with phospholipid headgroups on one side of the membrane was thinner. Estimating the thickness of that water layer as $d_{z}-d_{hh}-w_{h}/2$, and thus the total volume of water associated with each headgroup as $\left(d_{z}-d_{hh}-w_{h}/2\right)\times A_{c}$, and further assuming that a water molecule has a specific volume of $30\;{Å}^{3}$, we retrieve $n_{W}≃7$ for the POPC membrane (consistent with previous neutron scattering results [47]) and $n_{W}≃4.5$ for all the studied mito-like membranes.

Combining Equations (8) and (9) and using the fact that the integral of the unscaled profile over half the bilayer is zero, we have:

(11) $$\begin{array}{ccc} a & = & \frac{2\langle e_{c}\rangle }{A_{c}d_{z}}\;and\;b=\frac{\rho_{{\text{CH}}_{3}}-a}{\rho_{u}\left(0\right)} \end{array}$$

## 5. Conclusions

We have shown that, just as one-component or two-component membranes, the multi-component mitochondria-like phospholipid membrane which was the object of this study is subject to cholesterol’s condensation effect, albeit to a lesser degree. This suggests that the response of complex heterogeneous membranes, such as cellular membranes, to increase in cholesterol content is at least qualitatively similar to that of homogeneous model one-component membranes. Mitochondrial membranes are cellular membranes for which an elevated cholesterol content has drastic consequences, since it results in an inhibition of the recruitment and insertion of the pro-apoptotic protein Bax into in the membrane, and thus of Bax-mediated permeabilization, through a non-specific effect. Our study confirms that this must be due to one, or a combination, of the physical changes brought about by cholesterol condensation, namely: increase in chain order, thickening of the lipid bilayer and reduction in the average surface area per phospholipid chain.
